# Supplementary material for: Research on Physicochemical Properties and In Vitro Digestive Characteristics of High-Amylose Corn Starch–Ultrasound-Treated Waxy Rice Flour Blends
Source: Foods. 2025 Aug 21;14(16):2920. doi: 10.3390/foods14162920 (PMC12385470; doi:10.3390/foods14162920)
Supplement: Supplementary file 1 [file foods-14-02920-s001.zip › foods-3768322-supplementary.pdf]

Table S1. Effect of different levels of HACS addition on the relative crystallinity of HACS-UWRF blends.

HACS Addition/%	Relative crystallinity（%）
0	16.30±0.02[g]
5	16.90±0.11[f]
10	17.72±0.08[e]
15	18.49±0.18[d]
20	20.71±0.01[c]
25	21.69±0.06[b]
30	24.54±0.02[a]

Table S2 Effect of different levels of HACS addition on RDS, SDS and RS contents and eGI and HI values of rice cake.

HACS Addition/%	RDS(%)	RS(%)	SDS(%)	HI	eGI
0	23.77±0.46[a]	53.57±0.65[f]	22.65±0.23[d]	54.64±0.28[a]	69.16±0.15[a]
5	21.39±0.62[b]	53.99±0.24[f]	24.62±0.55[ab]	50.90±0.08[b]	67.66±0.04[b]
10	18.43±0.42[c]	56.54±0.55[e]	25.03±0.71[a]	48.41±0.32[c]	66.29±0.18[c]
15	17.43±0.24[d]	58.38±0.22[d]	24.18±0.05[abc]	45.84±0.10[d]	64.88±0.05[d]
20	15.95±0.05[e]	60.23±0.17[c]	23.82±0.19[abcd]	42.09±0.18[e]	62.82±0.10[e]
25	15.14±0.58[e]	61.50±0.11[b]	23.37±0.51[bcd]	39.07±0.07[f]	61.16±0.04[f]
30	13.16±0.63[f]	63.83±0.53[a]	23.01±0.14[cd]	35.06±0.03[g]	58.97±0.01[g]
